# Primary care visit trends in a public safety net health system during the COVID-19 pandemic

**DOI:** 10.3389/fpubh.2026.1808658

**Published:** 2026-05-26

**Authors:** Cristina Valdovinos, Yu-Chuang Huang, Stefanie Vassar, Griselda Gutierrez, Carmen Mendez, Anshu Abhat, Arleen Brown, Alejandra Casillas

**Affiliations:** 1 UCLA David Geffen School of Medicine, Los Angeles, CA, United States; 2 Division of General Internal Medicine and Health Services Research, UCLA David Geffen School of Medicine, Los Angeles, CA, United States; 3 Harbor-UCLA Medical Center, Los Angeles County Department of Health Services, Los Angeles, CA, United States

**Keywords:** digital divide, digital health equity, limited English proficiency, primary care, telehealth

## Abstract

**Background:**

Little is known about access to care and telehealth trends in safety net settings during the Coronavirus-19 (COVID-19) pandemic.

**Methods:**

We analyzed electronic health record data from patients assigned to a large, publicly funded, urban safety net system for primary care. We compared primary care visit types- in-person, telehealth (combined telephone and video visits), telephone, and video - between English-speaking patients and patients with limited English proficiency (LEP) across three study periods: pre-pandemic (March 2019–February 2020), pandemic Year 1 (March 2020–February 2021), and pandemic Year 2 (March 2021–December 2022).

**Results:**

Prior to the pandemic, 19% (English-speaking) and 17% (LEP) of primary care patients had a telehealth visit. This increased to 94% (English-speaking) and 95% (LEP) in Year 1 and 83% (English-speaking) and 85% (LEP) in Year 2. Over 80% of these telehealth visits were telephone visits.

**Conclusion:**

Telephone visits were critical in maintaining access to primary care during the COVID-19 pandemic. Eliminating telephone visits as a billable visit could disproportionately affect underserved populations.

## Background and significance

Telehealth, broadly defined by the Centers for Medicare and Medicaid Services (CMS) as the use of telecommunications and information technology to provide health care across distance, has the potential to expand health care access in safety net health systems (1). The implementation of telehealth in safety net health systems, settings that disproportionately serve racial/ethnic minority, low-income, and limited English proficiency (LEP) populations, as well as others who experience social barriers to health care, has historically faced significant challenges. Prior to the Coronavirus-19 (COVID-19) pandemic, CMS reimbursement policy restricted the delivery of telehealth services based on various geographic, provider, and visit characteristics. For example, patients were required to live in a rural or health care provider shortage area and travel to a medical facility to receive telehealth services and telephone encounters were not reimbursed (2).

In response to the COVID-19 public health emergency, CMS implemented flexibilities and regulatory requirement waivers that granted payment parity for telehealth, including both video and telephone visits, and allowed clinicians to provide telehealth services to patients in their homes (2). Prior studies have examined trends in ambulatory care visits during the COVID-19 pandemic and telehealth uptake patterns after the implementation of these policy changes, primarily among privately insured cohorts (3, 4).

However, little is known about telehealth utilization trends in safety net systems where the digital divide is well-established. Moreover, most prior studies in these settings have analyzed broad telehealth trends but have not distinguished between telephone and video visits. Examining trends by specific telehealth modalities is essential as vulnerable patients, including older adults and LEP patients, are less likely to use video visits (4). In one previous study, patients who were older, African-American, Medicaid beneficiaries, required an interpreter, or who lived in areas with low broadband access were less likely to use video visits than telephone visits (5).

The role of telephone visits as a mode of health care delivery during the COVID-19 pandemic remains underexplored. This is of particular concern given recent CMS changes in the reimbursement policies for telephone visits. As of 2026, telephone visits are eligible for Medicare reimbursement if the medical provider can use live video with the option of conducting a telephone visit if the patient cannot use video, a policy which may disadvantage safety net health systems that do not have the technology infrastructure to support video visits. Furthermore, Medicaid payment policies for telephone visits vary by state with some states limiting the telephone visits to certain specialties, e.g., mental health. As concerns over a worsening digital divide grow, understanding trends in telehealth utilization, particularly differences between video and telephone visit utilization, remains critical.

## Objective

The objective of this study is to describe and compare telehealth utilization trends among English-speaking and LEP patients across three phases of the COVID-19 pandemic in a large, urban safety net health system.

## Methods

We studied a large, publicly funded, urban safety net health system in Los Angeles comprised of major medical centers and an affiliated Ambulatory Care Network which provides over 2.5 million ambulatory visits annually.

To understand trends in telehealth utilization, we analyzed electronic health record data from patients assigned to this system for primary care. We used department codes in the electronic health record that correspond to primary care clinics to identify primary care visits. We defined telehealth as real-time, interactive, audio and/or video clinical encounters between a patient and a provider. In this study, the term telehealth refers to combined telephone and video visits.

Given the well-documented digital divide and barriers to video visits among vulnerable populations, we further categorized visits into telephone-only and video-only visits. We limited our analysis to primary care visits because telehealth access in specialty care settings remained far more limited during the study period. Additionally, primary care visits represent the most frequent point of contact for medically underserved and LEP patients in our health system.

We compared primary care visit types (in-person, telehealth, video, telephone) across three defined study periods: pre-pandemic (Mar 2019–Feb 2020), pandemic Year 1 (Mar 2020–Feb 2021) and pandemic Year 2 (Mar 2021-Dec 2022). Because nurse-only visits for COVID-19 vaccination increased significantly during the pandemic period resulting in an over-representation of in-person visits when compared to other clinical visits, these visits were excluded from these analyses. To account for seasonal fluctuations in visit volume across months (e.g., reduced visits during November and December presumably due to the traditional US holidays), raw visit counts were standardized and we calculated the number of primary care visits per 1,000 discrete patients served in primary care each month, stratified by visit modality (in-person, telehealth, telephone, and video).

Primary language was identified directly from the electronic medical record where it is recorded as part of patient registration. Patients were categorized into English-speaking or limited English proficiency (LEP). We paid special attention to primary language in our study because over half of visits in our system involve LEP patients. Since English as a primary language predicts telehealth utilization, we hypothesized that telephone and video visit use would differ between English-speaking and LEP patients. Therefore, we compared trends in visit types between patients with English-speaking and LEP patients across the three study periods.

The Chi-square tests for categorical variables (e.g., gender, race, ethnicity, ever had a telehealth visit) and t-tests for numerical variables (e.g., age, number of visits) were performed to test the statistical difference in demographic characteristics between English-speaking compared to LEP patients within each of the three study periods. All statistical analyses were conducted in SAS Statistical Procedures (6).

### Ethics statement

The study was reviewed and approved by the UCLA Institutional Review Board. Written informed consent for participation was not required for this study in accordance with the national legislation and the institutional requirements.

## Results

Table 1 shows the demographic characteristics of patients attending primary care visits compared across language groups. Across all study periods, LEP patients were more likely to identify as female (63.4–64.5%), Latino/Hispanic (88.1–88.9%), and were generally older (*p* < 0.001) than English-speaking patients. About 70% of patients in both language groups were Medicaid beneficiaries, i.e., participants in the California Medicaid program. LEP patients were more likely to be uninsured.

**Table 1:** Demographic characteristics of patients attending primary care visits in the Los Angeles County safety net across three study periods.

Variable	Pre-pandemic English (*n* = 79,193)	Pre-pandemic LEP^a^ (*n* = 79,978)	*p*-value	Pandemic Year 1 English (*n* = 76,555)	Pandemic Year 1 LEP^a^ (*n* = 76,052)	*p*-value	Pandemic Year 2 English (*n* = 80,313)	Pandemic Year 2 LEP^a^ (*n* = 81,082)	*p*-value
Gender			<0.0001			<0.0001			<0.0001
Female	41,475 (52.4%)	51,612 (64.5%)		40,380 (52.7%)	48,179 (63.4%)		42,256 (52.6%)	51,514 (63.5%)	
Race and Ethnicity			<0.0001			<0.0001			<0.0001
Asian	6,317 (8.0%)	4,576 (5.7%)		5,650 (7.4%)	3,881 (5.1%)		6,155 (7.7%)	4,148 (5.1%)	
Black	18,022 (22.8%)	258 (0.3%)		16,869 (22.0%)	203 (0.3%)		16,743 (20.8%)	203 (0.3%)	
Latino/Hispanic	27,268 (34.4%)	70,476 (88.1%)		26,932 (35.2%)	67,527 (88.8%)		28,809 (35.9%)	72,121 (88.9%)	
White	6,426 (8.1%)	1,039 (1.3%)		5,662 (7.4%)	855 (1.1%)		6,329 (7.9%)	978 (1.2%)	
Other Race	17,991 (22.7%)	2,966 (3.7%)		17,499 (22.9%)	2,668 (3.5%)		18,976 (23.6%)	2,805 (3.5%)	
Unknown Race	3,169 (4.0%)	663 (0.8%)		3,943 (5.2%)	918 (1.2%)		3,301 (4.1%)	827 (1.0%)	
Age			<0.0001			<0.0001			<0.0001
18–29	13,289 (16.8%)	4,065 (5.1%)		14,168 (18.5%)	3,133 (4.1%)		15,946 (19.9%)	3,115 (3.8%)	
30–49	26,238 (33.1%)	15,746 (19.7%)		26,338 (34.4%)	16,673 (21.9%)		28,234 (35.2%)	19,495 (24.0%)	
50–64	27,956 (35.3%)	36,599 (45.8%)		26,450 (34.6%)	35,982 (47.3%)		27,658 (34.4%)	39,304 (48.5%)	
65–74	10,136 (12.8%)	18,113 (22.6%)		8,215 (10.7%)	15,537 (20.4%)		7,217 (9.0%)	14,681 (18.1%)	
75+	1,574 (2.0%)	5,455 (6.8%)		1,384 (1.8%)	4,727 (6.2%)		1,258 (1.6%)	4,487 (5.5%)	
Mean (SD)	47.59 (15.65)	56.88 (13.80)	<0.0001	46.53 (15.47)	56.47 (13.19)	<0.0001	45.71 (15.30)	55.78 (12.88)	<0.0001
Insurance			<0.0001			<0.0001			<0.0001
Medicaid	54,713 (69.1%)	54,999 (68.8%)		53,749 (70.2%)	51,769 (68.1%)		58,522 (72.9%)	57,634 (71.1%)	
Medicare	7,216 (9.1%)	8,556 (10.7%)		6,887 (9.0%)	8,156 (10.7%)		7,181 (8.9%)	8,715 (10.7%)	
Private/commercial	12,014 (15.2%)	8,180 (10.2%)		10,248 (13.4%)	6,879 (9.0%)		10,804 (13.5%)	7,204 (8.9%)	
Uninsured	5,250 (6.6%)	8,243 (10.3%)		5,671 (7.4%)	9,248 (12.2%)		3,806 (4.7%)	7,529 (9.3%)	
Unhoused	268 (0.3%)	64 (0.1%)	<0.0001	596 (0.8%)	241 (0.3%)	<0.0001	509 (0.6%)	289 (0.4%)	<0.0001
Marital status			<0.0001			<0.0001			<0.0001
Married	15,210 (19.2%)	35,961 (45.0%)		13,910 (18.2%)	34,359 (45.2%)		14,813 (18.4%)	36,891 (45.5%)	

a LEP, limited English proficiency.

Table 2 summarizes primary care visit types (in-person, telehealth, telephone, and video) across the three study periods (i.e., pre-pandemic, pandemic Year 1 and pandemic Year 2). Before the pandemic, telehealth was utilized by around 19% of English-speakers and around 17% of LEP patients in this safety net system (Table 2). In-person visits declined during Years 1 and 2 while telehealth visits (combined telephone/video, telephone and video visits) for both language groups significantly increased. In Year 1, 94% of English-speakers and 95% of LEP patients had a telehealth visit. In Year 2, 83% of English-speakers and 85% of LEP patients in primary care had a telehealth visit. Most of the telehealth visits were telephone visits: over 90% of patients in both language groups had a primary care telephone visit in year 1 and over 80% had a primary care telephone visit in year 2. Video visits represented less than 1% of the telehealth visits in the health system across the three study periods.

**Table 2:** Primary care visit modalities (telehealth, telephone, video, in-person) across three study periods.

Variable	Pre-pandemic English (*n* = 79,193)	Pre-pandemic LEP^a^ (*n* = 79,978)	*p*-value	Pandemic Year 1 English (*n* = 76,555)	Pandemic Year 1 LEP^a^ (*n* = 76,052)	*p*-value	Pandemic Year 2 English (*n* = 80,313)	Pandemic Year 2 LEP^a^ (*n* = 81,082)	*p*-value
Type of visits									
Had a telehealth^b^ visit	14,627 (18.5%)	13,368 (16.7%)	<0.0001	71,809 (93.8%)	72,502 (95.3%)	<0.0001	66,328 (82.6%)	68,507 (84.5%)	<0.0001
Mean number of telehealth visits (SD)	0.25 (0.65)	0.22 (0.60)	<0.0001	3.44 (3.57)	3.94 (3.57)	<0.0001	2.33 (2.59)	2.50 (2.50)	<0.0001
Had a telephone^c^ visit	14,616 (18.5%)	13,363 (16.7%)	<0.0001	71,789 (93.8%)	72,490 (95.3%)	<0.0001	66,248 (82.5%)	68,455 (84.4%)	<0.0001
Mean Number of telephone visits (SD)	0.25 (0.65)	0.22 (0.60)	<0.0001	3.41 (3.48)	3.93 (3.55)	<0.0001	2.30 (2.53)	2.49 (2.49)	<0.0001
Had a video^d^ visit	14 (0.0%)	14 (0.0%)	0.979	825 (1.1%)	502 (0.7%)	<0.0001	884 (1.1%)	487 (0.6%)	<0.0001
Mean number of video visits (SD)	0.00 (0.01)	0.00 (0.02)	0.889	0.03 (0.48)	0.01 (0.21)	<0.0001	0.03 (0.46)	0.01 (0.18)	<0.0001
Had an in person visit	79,040 (99.8%)	79,928 (99.9%)	<0.0001	59,357 (77.5%)	64,101 (84.3%)	<0.0001	70,882 (88.3%)	75,892 (93.6%)	<0.0001
Mean number of in person visits (SD)	7.07 (7.45)	9.29 (8.61)	<0.0001	3.55 (5.11)	4.40 (5.84)	<0.0001	4.59 (5.63)	6.04 (6.64)	<0.0001
Had a primary care visit	79,193 (100.0%)	79,978 (100.0%)	—	76,555 (100.0%)	76,052 (100.0%)	—	80,313 (100.0%)	81,082 (100.0%)	—
Mean number of primary care visits (SD)	2.79 (2.12)	3.23 (2.23)	<0.0001	2.95 (2.55)	3.23 (2.47)	<0.0001	2.64 (2.03)	2.90 (2.00)	<0.0001

a LEP, limited English proficiency.

b Telehealth visit refers to telephone and/or video visit.

c Telephone visit refers to telephone visit only.

d Video visit refers to video visit only.

In the pre-pandemic period, English and LEP patients averaged 7.07 and 9.29 in-person ambulatory visits in the system, respectively. In-person visits declined substantially in pandemic Year 1 (English-speaking: 3.55; LEP: 4.40) and partially recovered in pandemic Year 2 (English-speaking: 4.59; LEP: 6.04). In the pre-pandemic period, both groups averaged fewer than one telephone or video visit. Telephone visits rose sharply in pandemic Year 1 (English: 3.41; LEP: 3.93) and declined in Year 2 (English: 2.30; LEP: 2.49) but remained higher than in pre-pandemic period, while video visits remained below one visit on average in both pandemic years. English-speaking and LEP patients averaged 2.79 and 3.23 total primary care visits, respectively, with no clinically significant changes across Year 1 or Year 2.

**Figure 1:**
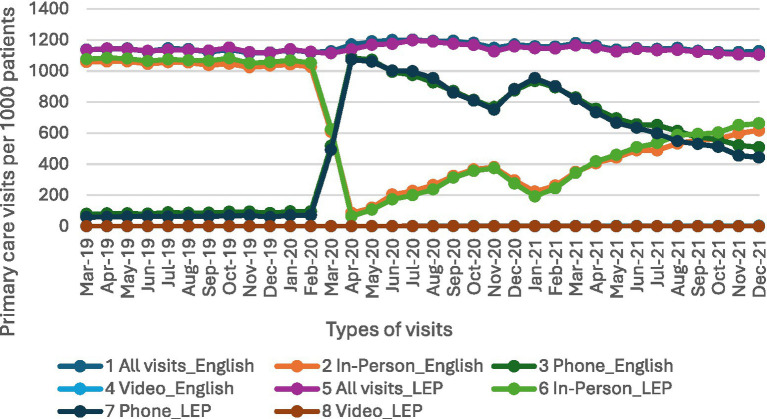
Monthly primary care visits per 1,000 patients by modality among English and LEP groups, from March 2019 to December 2021. LEP, limited English proficiency.

Figure 1 describes monthly primary care visits per 1,000 patients by primary care visit modalities (in-person, telehealth, telephone, video) among English and LEP groups between March 2019 and December 2021. The figure illustrates the sharp rise of telephone visits during this period in contrast to relatively stagnant video visit uptake across both language groups.

## Discussion

The results of this descriptive study demonstrate that this large, urban, publicly funded safety net system continued to deliver primary care visits during the COVID-19 pandemic, including to patients with limited English proficiency. Other studies of ambulatory care access in safety net centers including one study of 36 federally qualified health centers have reported a significant decline in total visits during the COVID-19 pandemic (7). In our study, while there was a modest decline in visit volume, per-patient primary care visit frequency remained relatively stable at around three visits per unique patient per year. Telehealth visits, particularly telephone visits, helped sustain continuity of primary care during the COVID-19 pandemic in our system.

Our study is one of the only studies of telehealth utilization in the safety net that categorized visits into in-person, telehealth, telephone-only, and video-only visits. Our results contribute to the existing evidence demonstrating that telephone visits are vital to preserving access to care for the underserved (5, 8). A study of over 100,000 patients at an academic medical center found that predictors of higher telephone visit use included older age, African American race/ethnicity, limited English proficiency, and lower income. A separate study of 45 health centers serving low-income populations found that telephone visits were used more frequently than video visits (8). Like this earlier work, our study suggests that for safety net patients, telehealth is synonymous with telephone visits. Contrary to our hypothesis, telephone and video visit use did not significantly differ across between English-speaking and LEP patients. Both language groups experienced a significant rise in telephone visits and minimal video visit uptake.

It is important to consider why telephone visit uptake is higher than video visit uptake among vulnerable groups. Underserved patients may have difficulty participating in video visits as these require both higher digital literacy skills and more broadband accessibility than telephone visits. Video visits generally require access via a patient portal and use of electronic interfaces that may not be optimized for the user’s preferred language or device (i.e., many developers forgo a mobile first design), and whose features may be too complex for the average user. Additionally, health centers in the safety net may lack the infrastructure to deliver timely, high quality video visits. As described in previous research, patient, clinic, and health system level factors influence telehealth implementation in safety net settings, and each of these factors must be addressed to optimize use and effectiveness of telehealth delivery (9).

A notable limitation of our study is the lack of generalizability to systems outside this safety net setting, as telehealth implementation remains highly system dependent. Our study examined telehealth utilization patterns in our system but did not examine health care quality and clinical outcomes. Additional research is needed on the quality of telehealth visits (e.g., care delivered, patient satisfaction) in comparison to in-person visits. Indeed, while telehealth has been associated with improved outcomes in the management of chronic diseases compared to in-person care, few studies have explicitly compared health outcomes across in-person, telephone, and video visits. Further research is needed in this area. This is an important next step of study within this safety net: to investigate the association between telephone and video visits with quality of care, particularly among patients with diabetes and hypertension, who make up a large proportion of this patient population. Additional limitations include the absence of a non-medically underserved comparison cohort and the single-state setting of our study, which may limit the generalizability of our findings to other health systems and geographic context.

The CMS waiver that allows payment parity for telehealth was recently extended until 2027; however, its future beyond this date remains uncertain. Our study suggests that telephone visits were critical in maintaining access to primary care during the COVID-19 pandemic. Although we have do not present information on billing for telephone visits and the possible impacts of reimbursement changes in this study, the results of this analysis highlight the importance of re-visiting the access implications for this waiver. Eliminating telephone visits as billable visits could disproportionately affect underserved patients and threaten the ability of safety net systems to maintain access to care for our nation’s most vulnerable patients, particularly in times of crisis like pandemics and natural disasters. While the use of video visits was very limited in our safety net setting, results of a national survey on telehealth utilization during the COVID-19 pandemic (4) showed that more than half of telehealth users reported use of video visits. Therefore, large reductions in payment rates for telehealth visits (including both telephone and video visits) in settings across the US could diminish access to ambulatory care for millions of marginalized Americans during public health emergencies and other large-scale crises. We recommend extending payment parity policies while more research is conducted on telemedicine’s effects on health outcomes, quality of care, and costs.

## Data Availability

The raw data supporting the conclusions of this article will be made available by the authors, without undue reservation.
